# Spatio-temporal variations in neonatal mortality rates in Ghana: An application of hierarchical Bayesian methods

**DOI:** 10.1371/journal.pgph.0000649

**Published:** 2022-09-08

**Authors:** Wisdom Kwami Takramah, Duah Dwomoh, Justice Moses K. Aheto

**Affiliations:** 1 Department of Epidemiology and Biostatistics, School of Public Health, University of Health and Allied Sciences, Ho, Ghana; 2 Department of Biostatistics, School of Public Health, University of Ghana, Accra, Ghana; Public Health Foundation of India, INDIA

## Abstract

Ghana might not meet the SDGs target 3.2 of reducing neonatal mortality to 12 deaths per 1000 live births by 2030. Identifying core determinants of neonatal deaths provide policy guidelines and a framework aimed at mitigating the effect of neonatal deaths. Most studies have identified household and individual-level factors that contribute to neonatal mortality. However, there are relatively few studies that have rigorously assessed geospatial covariates and spatiotemporal variations of neonatal deaths in Ghana. This study focuses on modeling and mapping of spatiotemporal variations in the risk of neonatal mortality in Ghana using Bayesian Hierarchical Spatiotemporal models. This study used data from the Ghana Demographic and Health Surveys (GDHS) conducted in 1993, 1998, 2003, 2008, and 2014. We employed Bayesian Hierarchical Spatiotemporal regression models to identify geospatial correlates and spatiotemporal variations in the risk of neonatal mortality. The estimated weighted crude neonatal mortality rate for the period under consideration was 33.2 neonatal deaths per 1000 live births. The results obtained from Moran’s I statistics and CAR model showed the existence of spatial clustering of neonatal mortality. The map of smooth relative risk identified Ashanti region as the most consistent hot-spot region for the entire period under consideration. Small body size babies contributed significantly to an increased risk of neonatal mortality at the regional level [Posterior Mean: 0.003 (95% CrI: 0.00,0.01)]. Hot spot GDHS clusters exhibiting high risk of neonatal mortality were identified by LISA cluster map. Rural residents, small body size babies, parity, and aridity contributed significantly to a higher risk of neonatal mortality at the GDHS cluster level. The findings provide actionable and insightful information to prioritize and distribute the scarce health resources equitably to tackle the menace of neonatal mortality. The regions and GDHS clusters with excess risk of neonatal mortality should receive optimum attention and interventions to reduce the neonatal mortality rate.

## Introduction

The major problem that has confronted health managers and administrators in recent years has to do with equitable allocation of scarce human and health resources to the needy areas. Identification of areas with excess risk or hot spots and the degree of spatial clustering and dispersion in health events can inform efficient distribution and utilization of scarce health resources.

Several epidemiological and public health studies on disease mapping focus on small area estimation where the dependent variable of interest such as disease incidence or mortality and corresponding risk factors are aggregated at the areal unit level including regional, district, sub-district and enumeration area levels. Standardized mortality ratio (SMR) is a good measure of disease or mortality risk [1,2].

Neonatal mortality is a major global health problem. A review of the literature indicates that 2.6 million newborns die every year within 28 days of life in the world. Unfortunately, one million of the newborn babies who do not survive beyond 28 days of life die on the very day they are born [3]. Sustainable Development Goal (SDG) 3 seeks to put an end to preventable neonatal deaths and under-five deaths, and all countries are tasked to reduce neonatal mortality to 12 deaths per 1000 live births and under-five mortality to 25 deaths per 1000 live births by 2030[4–6]. However, Ghana is nowhere close to the SDGs target 3 of reducing neonatal mortality to 12 deaths per 1000 live births. Ghana has been grappling with high neonatal mortality risk for decades.

Even though the Demographic and Health Survey data for all survey rounds depicted regional and temporal [7,8] disparity in neonatal mortality in Ghana, no study has been conducted to fit spatiotemporal model to estimate and map spatiotemporal pattern of relative risk of neonatal mortality in Ghana. We seek to bridge this gap in the existing literature using the sophisticated statistical methods at our disposal: the Bayesian Hierarchical Spatiotemporal model that accounts for both the spatial and temporal variations in neonatal mortality. The findings from this study will guide the stakeholders in the health sector in formulating and implementing appropriate policies focused on preventing neonatal deaths. This study sought to quantify the extent of geospatial clustering of the risk of neonatal deaths at the regional and GDHS cluster levels in Ghana and how they vary over time.

## Materials and methods

### Study population

The objectives of the current study require the merging and use of 1993, 1998, 2003, 2008 and 2014 Ghana Demographic and Health Surveys (GDHS) datasets extracted from the Monitoring and Evaluation to Assess and Use Results (MEASURE) program database. The shapefiles used for the base layers of the maps were downloaded from the DHS program website upon registration and request [9]. Detailed information on terms of use of the shapefiles (GPS datasets) can be accessed at the DHS program website[10,11]. The GDHS rounds are nationally representative household sample surveys and the sampling frames used were obtained from the 1984, 2000, and 2010 Ghana Population and Housing Census (PHC) obtained from the Ghana Statistical Service. In Table 1, the total number of enumaration areas (EAs) or clusters selected for 1993, 1998, 2003, 2008 and 2014 GDHS were 400, 400, 412, 412 and 427 respectively.

**Table 1:** Summary of enumeration areas and the number of successfully completed interviews for all rounds of Ghana demographic and health surveys.

Survey Year	No. of EAs	Successfully Completed Interviews					
		Households	Women age (15–49)		Men age (15–49)		Children under 5 years
			Urban	Rural	Urban	Rural	
**GDHS1993**	400	5822	1720	2842	640	842	2204
**GDHS1998**	400	6003	1,585	3,258	492	1,054	3298
**GDHS2003**	412	6,251	2,374	3,317	1903	3112	3844
**GDHS2008**	412	11,778	2,162	2,754	1,914	2,654	2992
**GDHS2014**	427	12150	5098	5116	2061	2114	5884

EAs = Enumeraiton areas.

Source: Ghana Statistical Service.

Information gathered from Ghana Statistical Service (GSS), indicated that the 1993 GDHS successfully interviewed 4,562 women aged 15–49 years and 1,302 men aged 15–59 years drawn from 6000 households whilst the 1998 GDHS successfully completed interviews for 6,003 households, 4,843 eligible women aged 15–49 years and 1,546 eligible men aged 15–59 years. Additionally, the 2003 GDHS selected and successfully interviewed 5,691 women aged 15–49 years and 5,015 men aged 15–59 years from 6,251 households in Ghana (Table 1). A total of 4,916 women aged 15–49 years and 4,568 men aged 15–59 years from 6,141 households were selected and interviewed using women and men questionnaires respectively while with the household questionnaire, a total of 11,778 households were interviewed for 2008 GDHS [12]. The 2014 GDHS successfully interviewed 11,385 occupied households of which 9,396 women aged 15–49 years were interviewed and 4,388 identified men aged 15–59 years were successfully interviewed.

Two-stage sampling technique was the survey design used in the GDHS of all survey rounds where the enumeration areas (Primary Sampling Units) were selected within each rural and urban location and the second stage involved the use of systematic sampling technique to select households (Secondary Sampling Units) from each cluster (EAs) at random.

Since the focus of the current study was on neonatal deaths, we reviewed the birth history data collected using the children under 5 year’s questionnaire which obtained data on 2204, 3298, 3844, 2992 and 5884 children born in the five years preceding the 1993, 1998, 2003, 2008 and 2014 GDHSs respectively.

The data underlying our analysis is freely available at the DHS website (https://dhsprogram.com/data/available-datasets.cfm).

### Outcome variable

The number of neonatal deaths aggregated at the regional and GDHS cluster levels in the five years preceding the various GDHS survey rounds was the primary outcome measure of interest.

### Independent variables

The covariates were selected based on the conceptual framework for the study of child survival in developing countries developed by Mosley and Chen [13], and other previous studies[8,14–16]. The covariates considered for analysis were the sex of child, birth type, birth interval, health insurance status of mother, weight of the child, maternal age, ever terminated pregnancy, mode of delivery, maternal marital status, number of children ever born, household size, maternal religion, literacy of mother, babies delivered at home, babies breastfed within 24 hours of life, unimproved sanitation facilities, unimproved water source facilities, delivery care, wealth index, prenatal care assistance and time to reach water source and return. Other climate covariates such as average maximum temperature, malaria prevalence, and aridity, diurnal temperature, and precipitation were also included in the variable selection models.

### Statistical analysis

The required GDHS data for 1993, 1998, 20003, 2008 and 2014 survey rounds were merged and transferred to RStudio version 1.4.1106 for analysis. Data pre-processing and cleaning were done in RStudio version 1.4.1106 and Stata version 15 to remove observable noise in the data. Data consistency and duplicate checks were performed to render quality data for analysis. The study variables were checked for any possible data collection and entry errors. The categorical variables were checked to ensure that all the categories were mutually exclusive and exhaustive. The quantitative variables were also checked for outliers using a box plot and histogram.

The survey design characteristics such as clustering, stratification and sampling weights were incorporated in the models. According to the DHS statistics guide, all forms of data generated from DHS must be weighted. This is due to data complexity as a result of survey design characteristics such as unequal probability of sample selection, clustering and stratification. Hence, adjustment factor such as sampling weights, clustering and stratification were applied to each case in tabulation to adjust for differences in the probability of selection and interview between cases in a sample. Furthermore, this weight was applied in tabulation to produce the appropriate representation and correct parameter estimate. The sampling weights applied to each sampling unit (k) was used to obtain the weighted estimate of neonatal deaths.

Exploratory analysis was carried out by computing and describing neonatal mortality rates and other relevant indicators for all the aforementioned GDHS survey years. The distribution of the number of deaths and relative risk (RR) were displayed on a map for clear and easy visualization of spatial and temporal variations in the areal count and point level data.

To observe clustering of relative risk of neonatal mortality among GDHS clusters, the data were aggregated at the cluster level. Since the GDHS clusters present point level data. Integrated Nested Laplace Approximation (INLA) framework was used to fit the Bayesian geospatial model via stochastic partial differential equation (SPDE). The INLA which is more computationally convenient and efficient than other parameter estimation methods such as MCMC methods was applied to the spatial and spatiotemporal areal count data to estimate posterior distribution of the parameter of interest. INLA uses numerical integration algorithm for sparse matrices to approximate posterior marginal distribution of the model parameters [2].

Moran’s I statistics was calculated to assess both global and local autocorrelation (spatial clustering) using a binary distance matrix. To achieve this, sets of neighbors were created based on their proximity. A normality check on the relative risk of neonatal mortality revealed a skewed distribution. Thus, the relative risk of neonatal mortality was transformed using natural logarithm because the Moran’s I test is best for normally distributed data. A spatial test for clustering was performed by assigning row standardized binary weights to the neighbor list, using minimum distance for one neighbor. Since Global Moran’s I statistic does not provide information on the location of clusters, we assessed the existence of local clustering using local indicator of spatial association (LISA) and the points were mapped to display significant local clusters. A LISA is an important tool for examining location of the clusters because it provides a statistic for each location with an assessment of significance [17].

### Model formulation

Geospatial analysis considered for the current study was centered on disease (mortality) mapping across regional level *i* and at time *t* (survey rounds), all the relevant variables were aggregated across 10 areal units (regions). To this end, the number of neonatal deaths *Y*_*it*_ observed in region *i*, *where i* = 1,2,..,10 and time period *t*, *where t* = 1,…,5 resulted in a 10×5 matrix. INLA framework was used to fit Bayesian Hierarchical model via conditional autoregressive (CAR) model and Gaussian or Independent and identically distributed (iid) model. The CAR model was specified to account for autocorrelation (spatial clustering) by assessing whether the areal data depicted a spatial structure such that observations from neighboring regions exhibited higher correlation than regions far apart [18,19].

The uninformative priors on the log of the precision of the hyper-parameters included *τ*_*μ*_~logGamma (1, 0.00005) for structured spatial random effect and *τ*_*v*_~*logGamma* (1,0.00005) for unstructured spatial random effect [20]. The prior on the precision of unstructured temporal effect included *τ*_∅_~*logGamma*(1,0005) and the prior specification of *τ*_*γ*_~*logGamma*(1,001) on the precision of random walk of first order (RW1) was used [20].

The general spatiotemporal models for areal unit data when time is discrete were proposed by spatial statisticians [1,17,21]. Let *Y*_*it*_ denote number of observed neonatal deaths in region *i* and time period t, *E*_*it*_ denotes expected number of neonatal deaths in region *i* and time period *t*, *n*_*it*_ denotes the number of persons at risk at region *i* in year *t*.

The expected number of neonatal deaths in region *i* and time period *t* is defined as:

$$E_{it}=n_{it}\frac{\sum_{it}Y_{it}}{\sum_{it}n_{it}}$$ (1)

The formula for estimating relative risk (*θ*_*it*_) of mortality in each areal unit *i* at time period *t* is given as:

$${\hat{\theta }}_{it}=\frac{Y_{it}}{E_{it}}$$ (2)

The focus of modeling is the log relative risk;

$$\log\left(\theta_{it}\right)=\psi_{it}$$

Thus, the model for neonatal mortality count *Y*_*it*_ observed in region *i* at time period t is defined as a three-level Bayesian Hierarchical model (BHM):

Data model:

$$Y_{it}|\psi_{it}∼Poisson(E_{it}e^{\psi_{it}}),$$ (3)

where *ψ*_*it*_ denotes log-relative risk of neonatal mortality for region *i* at time period t and *E*_*it*_ denotes expected number of neonatal mortality in region *i* at time period t.

The Bayesian Hierarchical Spatiotemporal models of log-relative risk which compose of spatial and temporal structures were proposed by many spatial statisticians [21–23]:

$$Process\;model:\;\psi_{it}=\alpha +\mu_{i}+\upsilon_{i}+\gamma_{t}+\emptyset_{t}$$ (4)

Parametermodel:υiiid∼N(0,τυ−1)

$$\mu_{i}|\mu_{j\neq i}∼CAR(W,\tau_{\mu }^{-1})$$

$$\gamma_{t}|\gamma_{t-1}∼N(\gamma_{t-1},\tau_{\gamma }^{-1})$$

$$\emptyset_{t}∼N(0,\tau_{\emptyset }^{-1})$$

nowτViid∼Gamma(1,0.00005)andτμiid∼Gamma(1,0.00005)

τγiid∼Gamma(1,001)andτ∅iid∼Gamma(1,0.00005),

where *α* denotes the intercept term, *μ*_*i*_ denotes structured spatial random effect or correlated heterogeneity effect (CH) which were assigned CAR model, *υ*_*i*_ denotes unstructured exchangeable spatial component that models heterogeneity or uncorrelated heterogeneity effect (UH) among the locations at time t which is independently and identically distributed. The correlated heterogeneity effect *μ*_*i*_ and uncorrelated heterogeneity effect *υ*_*it*_ are spatial random effects which can vary in time. *γ*_*t*_+∅_*t*_ is temporal main random effects. *γ*_*t*_ is specified using autoregressive prior distribution and can follow a random walk in time of first order (*γ* = 1) which allows for non-parametric temporal effect. Whereas ∅_*t*_ represents unstructured temporal effect which is independently and identically distributed.

The Besag York Mollie model [23–25] defined above could be extended to include covariates:

$$\psi_{it}=\alpha +{X_{it}^{\prime }\beta +\mu }_{i}+\upsilon_{i}+\gamma_{t}+\emptyset_{t}$$ (5)

Where $X_{it}^{\prime }$ is a matrix of covariates, ***β*** denotes unknown corresponding parameter vector coefficients.

The weighted Bayesian Hierarchical Spatiotemporal model without covariates (model 1) and weighted Bayesian Hierarchical Spatiotemporal model with covariates (model 2) were evaluated using Log-pseudo marginal likelihood (LPML) [26]. Log-pseudo marginal likelihood (LPML) also known as logarithm score (log-score) is useful for non-linear and mixture models with discrete parameters [22] and the number of random effect components in the model. Thus, the model with the largest Log-pseudo marginal likelihood was chosen as the best model to fit the data. The Log-pseudo marginal likelihood (LPML) is an example of leave-one-out cross-validation score which is an excellent model evaluation tool for hierarchical complex models with many random effects. Other model evaluation metrics such as Deviance Information Criteria (DIC) and Watanabe-Akaike Information Criterion (WAIC) were computed.

The selected model estimated the posterior mean of relative risk of neonatal mortality and investigated whether a unit increase of the covariates increases or decreases the value of relative risk of neonatal mortality. Thus, the estimated Bayesian credible intervals were used to examine the significance of the covariates included in the models.

The model for neonatal mortality count *y*_*i*_ observed in GDHS cluster *i* is defined as a three-level Bayesian Hierarchical model (BHM):

$$Data\;model:\;y_{i}|\psi_{i}∼Poisson(E_{i}e^{\psi_{i)}},$$ (6)

$$\log(\theta_{i})=\psi_{i}=\alpha +x_{i}\beta +\mu_{i}+\upsilon_{i},i=1,2,\ldots ,p$$ (7)

$$\alpha ∼N(0,\tau_{0}^{-1})$$

$$\upsilon_{i}∼N(0,\tau_{\upsilon }^{-1})$$

$$\mu_{i}|\mu_{j\neq i}∼N({\bar{\mu }}_{i},\frac{1}{\tau_{\mu }m_{i}}),$$

$${\bar{\mu }}_{i}=m_{i}^{-1}\sum_{j\in \partial_{i}}\mu_{j}$$

$$\tau_{v}∼Gamma(a,b)$$

$$\tau_{\mu }∼Gamma(c,d)$$

Where *y*_*i*_ is count of disease/mortality in area *i*, *E*_*i*_ denotes the standardized expected rate in area *i* and *ψ*_*i*_ denotes the log relative risk in areal *i*, *x*_*i*_***β*** denotes a trend or fixed covariate component, *x*_*i*_ denotes vector of the intercept and *p* covariates per area unit *i*, and ***β*** denotes vector of regression parameter, *μ*_*i*_ are denote structured spatial random effect (correlated heterogeneity) and *υ*_*i*_ denotes unstructured spatial random effect (uncorrelated heterogeneity), ${\bar{\mu }}_{i}$ is the mean of the neighboring *μ*_*j*_ values, *m*_*i*_ denote the number of neighbors of area *i*, $\frac{1}{\tau_{\mu }m_{i}}$ denote the variance for area *i*, *τ*_*μ*_ denotes precision parameter which controls the degree of smoothness or extra-Poisson variability assigned to clustering *μ*_*i*_, *τ*_*v*_ denotes a precision parameter that controls the magnitude of the *υ*_*i*_. All parameters such as *α*, *μ*_*i*_, *υ*_*i*_, *τ*_*v*_, *τ*_*μ*_ are assigned prior distributions in the Bayesian modelling paradigm. The spatially structured random effects (*μ*_*i*_) were assigned CAR distribution.

### Ethics approval

The 2014 GDHS protocol was reviewed and approved by the Ghana Health Service Ethical Review Committee and the Institutional Review Board of ICF International. Thus, ethical permission for the current study was not needed since the required GDHS dataset was available publicly. However, ethical procedures were strictly observed at the time of the GDH surveys to ensure that human rights were protected as required by US Department of Health and Human Services. Detail information on DHS data and ethical standards can be accessed at: http://goo.gl/ny8T6X.

## Results

Table 2 presents global spatial autocorrelation coefficients computed using aggregated GDHS cluster level data for each GDHS survey year. The p-values computed for Global Moran’s I tests under the null hypothesis of spatial randomness indicated existence of significant spatial clustering but slightly weak autocorrelation. The existence of clustering justifies the use of Bayesian Hierarchical Spatial and Spatiotemporal model to smooth the neonatal data. Thus, maps were generated to depict spatial clustering of neonatal mortality.

**Table 2:** Testing for global spatial autocorrelation using Moran’s I statistics for each survey year.

Year	Moran’s I Statistics	P-value
1993	0.085	0.001
1998	0.134	0.001
2003	0.222	0.001
2008	0.072	0.001
2014	0.333	0.001

### Summary statistics of selected indicators by GDHS survey years

**Fig 1:**
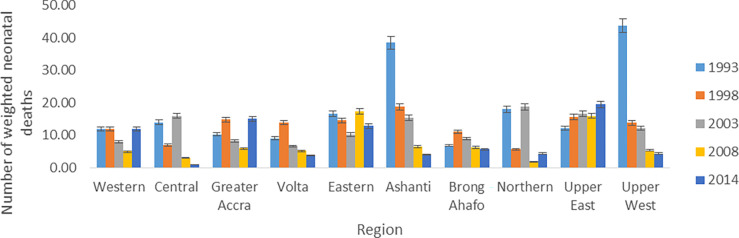
A barchart showing the regional trends in weighted observed neonatal mortality across DHS survey years in Ghana. Note: We applied survey weights in our estimation hence the term weighted observed neonatal mortality.

Fig 1 shows a rapid decline in neonatal mortality in Ashanti and Upper west regions over the survey years under consideration. In contrast, Upper East region saw a marginal increase in neonatal mortality across the survey years. It is evident that, the observed neonatal mortality declined steadily from 1998 to 2014 in Volta and Brong Ahafo regions. However, extremely high observed neonatal deaths were recorded in Ashanti and Upper west regions in 1993.

Table 3 displays point estimates of key indicators of neonatal mortality. The weighted SMR estimated for the survey years indicated that a high risk of neonatal mortality was recorded in 1993 (SMR = 1.08), 1998 (SMR = 1.16) and 2014(1.05). It is notable that a high relative risk of neonatal mortality was recorded in 1993(RR = 1.12) and 1998 (RR = 1.02). Additionally, the estimated overall weighted crude neonatal mortality ratios for the ten regions across the 5 survey years considered in the current study was 33.19 per 1000 live births.

**Table 3:** Distribution of selected indicators in Ghana (1993–2014).

Indicator	Year				
	1993	1998	2003	2008	2014
Weighted SMR	1.08	1.16	0.95	0.82	1.05
Weighted NMR (per 1000 live births)	40.47	29.24	39.64	29.68	28.50
Weighted relative risk	1.12	1.02	0.89	0.83	0.95
Combined weighted crude neonatal mortality ratio (per 1000 live births)	33.19				

### Variable selection

The covariates were aggregated at the regional level and GDHS cluster level. Of the candidate covariates considered in the current study, the Akaike Information Criterion (AIC) score favored 11 covariates (number of small body size baby, Median household size, median parity, Proportion of poor mothers, Number of rural dwellers, Proportion of unimproved sanitation facilities, median time to reach water source and return, Median Maximum Temperature, Median Aridity Median, land surface temperature, median diurnal temperature). Additionally, multicollinearity test was performed to assess the existence of collinearity between the covariates by setting a threshold of 10. Thus, covariates with the score of variance inflation factor (VIF) greater than 10 were removed from the models.

### Model selection and validation

Table 4 depicts the evaluation of 2 models including the Bayesian Hierarchical Spatiotemporal model without covariates (model 1) and Bayesian Hierarchical Spatiotemporal model with covariates (model 2) using model selection tools such as DIC, WAIC and LPML. The best model was chosen based on the LPML (log-score) score. A larger value of the LPML score indicates a better prediction quality of the model. Thus, a larger LPML score of 144.67 favored model 2. It is notable that the smaller DIC (275.86) and WAIC (283.96) scores also favored model 2.

**Table 4:** Evaluation of Bayesian Hierarchical Spatiotemporal model without covariates (model 1) and Bayesian Hierarchical Spatiotemporal model with covariates (model 2).

	DIC	WAIC	LPML
Model 1	284.14	286.85	143.48
Model 2	275.86	283.96	144.67

### Quantifying the extent of geospatial clustering of the risk of neonatal deaths at the regional level in Ghana and how they vary over time

**Fig 2:**
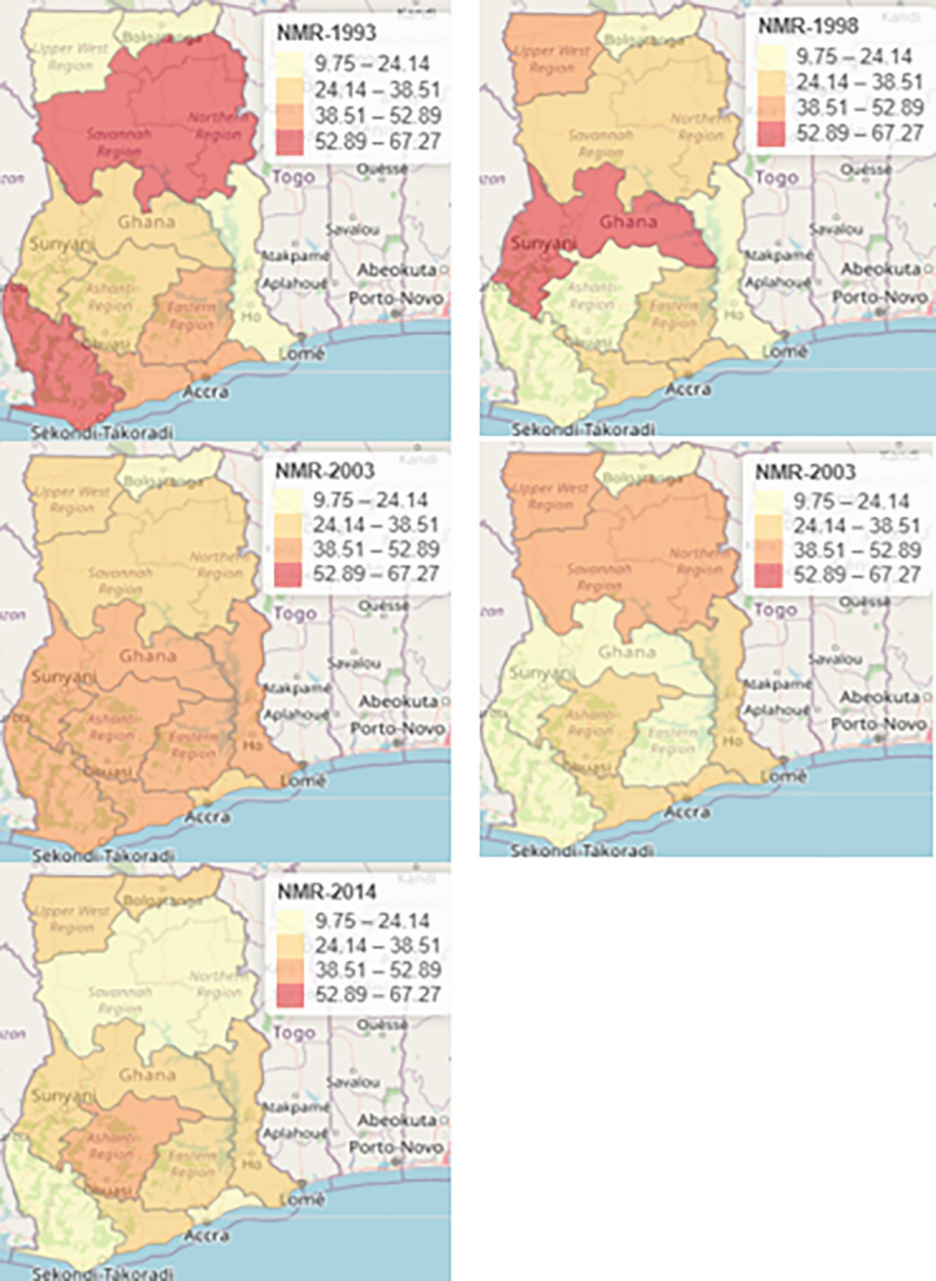
Map displaying weighted neonatal mortality rates for the ten regions in Ghana, 1993–2014. Note: This map was produced by the authors.

In Fig 2, the map illustrates weighted neonatal mortality rates in the then 10 regions in Ghana. To support better visualization and comparison, we fixed the same scale for the weighted neonatal mortality rates for all the 5 survey years. It is notable in the map for 1993 that a cluster of low neonatal mortality rate was recorded in Upper West, Upper East and Volta regions and a high neonatal mortality rates was estimated in Northern and Western regions. In 1998, a moderately high neonatal mortality rate was recorded in Upper West region and a high neonatal mortality rate was observed in the Brong Ahafo region. A cluster of moderately high neonatal mortality rate was identified in Brong Ahafo, Eastern, Central, Ashanti, Volta and Western regions in 2003. Geographical areas such as Upper West and Northern regions reported a moderately high neonatal mortality rate in 2008. Furthermore, a moderately high neonatal mortality rate was observed in the Ashanti in 2014. A consistently low neonatal deaths was identified in Upper East in 1993, 1998, 2003, 2008 and slightly inched up in 2014.

**Fig 3:**
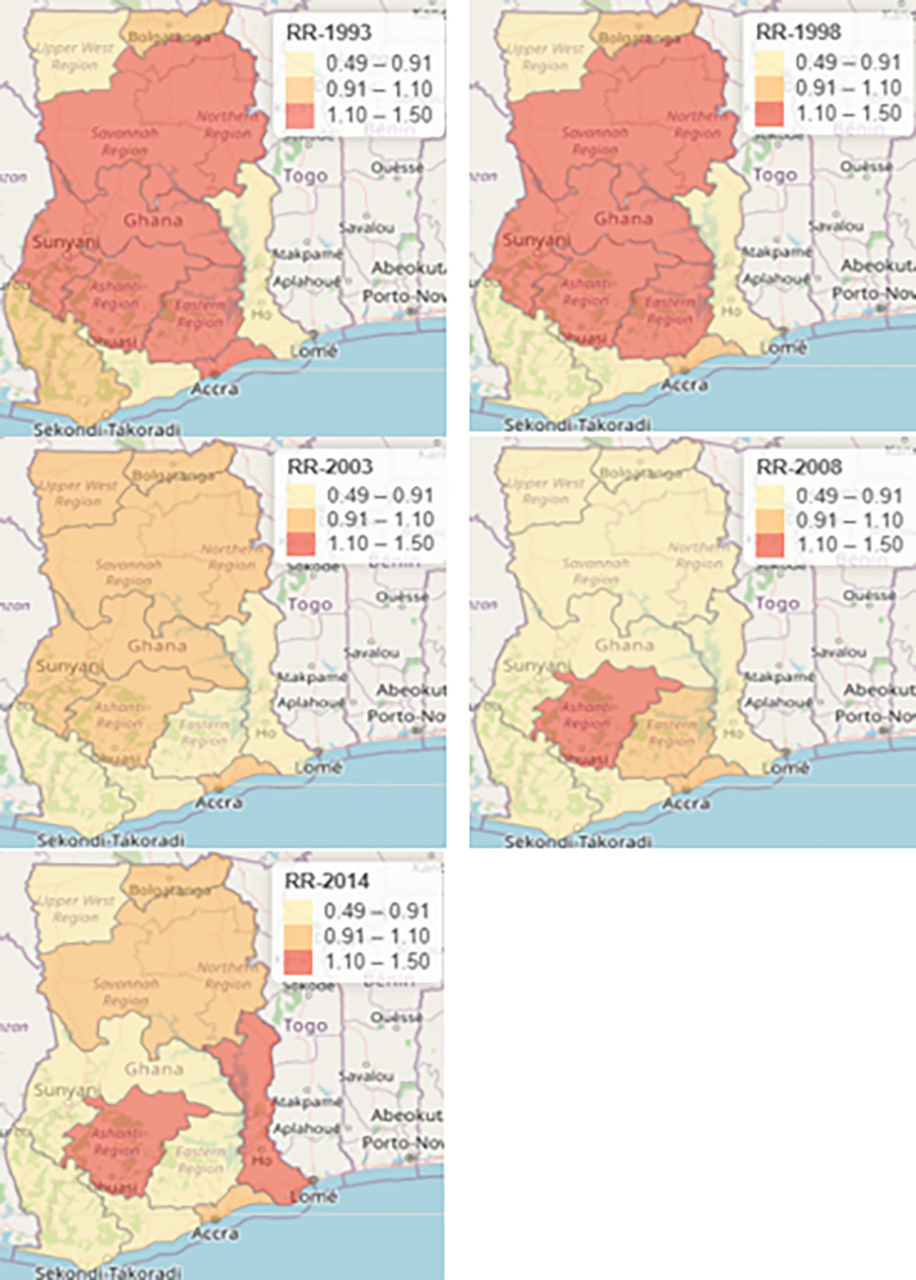
Map showing smooth relative risk of neonatal deaths across the then 10 regions in Ghana obtained from weighted Bayesian Hierarchical Spatiotemporal model with covariates, 1993–2014. Note: This map was produced by the authors.

Fig 3 shows a map of predicted or smooth relative risk of neonatal mortality estimated from the weighted Bayesian Hierarchical Spatiotemporal model for each survey years. Again, to support better visualization and comparison, we fixed the same scale for the relative risk of neonatal mortality for all the 5 survey years. A cluster of a high relative risk of neonatal mortality was observed in the Northern, Ashanti, Brong Ahafo, Eastern, Central, and Greater Accra regions in 1993. Furthermore a cluster of high relative risk was recorded in neighboring regions such as Ashanti, Eastern, Brong Ahafo and Northern regions in 1998. It is observed that the relative risk of neonatal mortality estimated in Greater Accra, Brong Ahafo, Upper West, Upper East, Northern and Ashanti regions was not different from the risk that we would have expected in the standard population in 2003. A high relative risk of neonatal deaths was estimated in Ashanti in 2008. Similarly, the relative risk observed in Eastern regions was the same as the expected risk in the standard population in 2008. Geogrpical areas such as Volta and Ashanti regions shared the same high risk of neonatal deaths in 2014. A cluster of low relative risk was identified in Brong Ahafo, Western, Central and Eastern regions in 2014

### Identifying correlates associated with the risk of neonatal mortality clustering at regional level

Table 5 shows that the number of small body size babies was the only significant covariate in model 2. It is notable in model 2 that the posterior mean of the number of small body size baby estimated from the Bayesian Hierarchical Spatiotemporal model indicated a marginal increase in the risk of neonatal deaths [PM: 0.003 ((95% 0.00,0.01)] with an estimated relative risk of 1.003 [exp(0.003)]. All geographical factors in model 2 were not significant.

**Table 5:** Summary of posterior means and Bayesian credible intervals of smooth relative risk of neonatal mortality estimated from weighted Bayesian Hierarchical Spatiotemporal model with and without covariates using aggregated 1993–2014 Ghana demographic and health survey (GDHS) regional level data.

Parameters	Model 1	Model 2
	Posterior Mean(95% Cr.I)	Posterior Mean(95% Cr.I)
***Fixed Effect***		
*Intercept*	-0.048 (-0.14, 0.04)	-0.13(-2.24, 2.03)
***individual factors***		
*small body size baby*		0.003(0.00,0.01)
*Median household size*		-0.14(-0.32, 0.04)
*Proportion of babies who were breastfed within 24hrs of life*		-0.39(-1.24, 0.45)
*median parity*		0.13(-0.17, 0.43)
***Community factors***		
*median time to reach water source and return*		0.004(-0.03, 0.02)
***Geographical factors***		
*Median Aridity*		0.03(-0.02, 0.08)
*Median precipitation*		0.01(-0.003, 0.02)
Median diurnal temperature		-0.03(-0.19, 0.12)
*Median land surface temperature*		-0.03(-0.08, 0.03)
**Random Effect**		
$\tau_{\mu }^{-1}$	25873.14(2448.85, 99521.56)	21761.19(1833.40, 79892.70)
$\tau_{\upsilon }^{-1}$	18313.65(1161.38, 67732.92)	30060.28(2790.80, 106739.79)
$\tau_{\gamma }^{-1}$	114.10(15.52, 371.67)	123.64(14.50, 401.38)
$\tau_{\emptyset }^{-1}$	25411.12(2372.27, 97632.50)	24601.21(2283.44, 93704.41)
**Model Evaluation Metrics**		
DIC	284.14	275.86
WAIC	286.85	283.96
LPML (Log score)	143.48	144.67

Model 1 = Bayesian Hierarchical Spatiotemporal model without covariates (empty model), Model 2 = Bayesian Hierarchical Spatioemporal model with covariates, DIC = Deviance Information Criterion, WAIC = Watanabe–Akaike information criterion, LPML = Log Pseudo Maximum Likelihood.

With reference to model 2, there was spatial dependence or spatial autocorrelation in the neonatal mortality because the 95% credible interval associated with the posterior mean of the precision of structured spatial random effect (CAR) $\tau_{\mu }^{-1}$ shows some spatial dependence [Posterior Mean: 21761.19 (95% CrI: 1833.40, 79892.70)]. The posterior mean of $\tau_{\upsilon }^{-1}$ [Posterior mean: 30060.28(95% CrI: 2790.80, 106739.79)] indicated that most of the variations in the risk of neonatal mortality were explained by the intercept term *α* and the covariates in model 2 relative to model 1 [Posterior mean: 18313.65(95% CrI: 1161.38, 67732.92)].

The estimated posterior mean of the precision of structured temporal random effect (RW1) $\tau_{\gamma }^{-1}$ shows some temporal dependence dependence [Posterior Mean: 123.64 (95% CrI: 14.50, 401.38)]. Both spatial and temporal random effects are significant.

### Quantifying the extent of geospatial clustering of the risk of neonatal deaths at the GDHS cluster level in Ghana and how they vary over time

**Fig 4:**
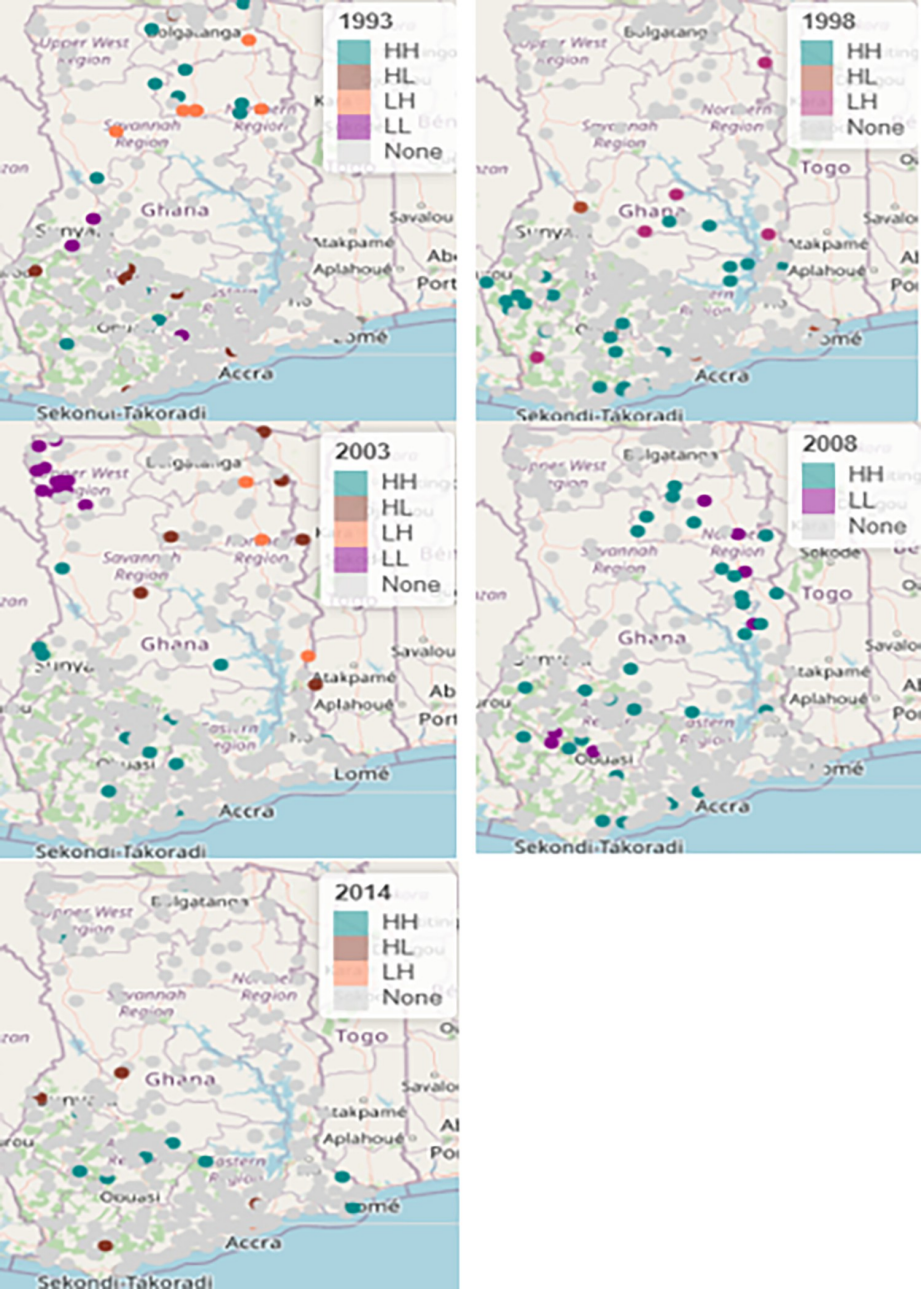
LISA Cluster map showing relative risk of neonatal mortality across GDHS clusters with associated geographical, individual and community factors, 1993–2014. Note: This map was produced by the authors.

A LISA cluster map in Fig 4 displays five types of geographical clustering such as high-high spatial clusters (hot spot), Low-Low spatial clusters (cold spot), high-low spatial outliers, low-high spatial outliers, and non-significant spatial points. A GDHS cluster with a significantly high value of the relative risk of neonatal mortality surrounded by its neighbors with a significantly high relative risk of neonatal mortality were observed in Northern, Upper West and some part of Western and Ashanti regions respectively in 1993 giving rise to High-High clusters. Hot spots GDHS clusters were observed in Central, Western, Volta, Brong Ahafo, Eastern and Ashanti regions while a few spatial outliers (overdispersion) of risk of neonatal mortality for some GDHS clusters were recorded in Ashanti, Brong Ahafo and Northen regions in 1998. It is observed in 2003 that a significantly high relative risk of neonatal mortality estimated for some GDHS clusters correlated with a high risk of neonatal mortality for neighboring GDHS clusters in Ashanti, Brong Ahafo and Northern regions compared to low-low neonatal mortality risk for neighboring GDHS clusters in Upper East and Upper West regions. The cluster map also depicts hot spot GDHS clusters in Ashanti, Northern and the coastal areas while clusters of a low relative risk of neonatal mortality estimated for GDHS clusters were observed in the Upper west and the Northern regions in 2008. It is notable that a GDHS cluster with a significantly high relative risk was surrounded by its neighbors with a high relative risk in Ashanti, Eastern and Volta regions in 2014. A GDHS cluster of a high relative risk of neonatal mortality and a GDHS cluster of a low neonatal mortality was also observed in Brong Ahafo, Ashanti, Eastern and Central regions in 2014.

### Identifying correlates associated with the risk of neonatal mortality clustering at the DHS cluster level

Table 6 presents a summary of posterior means and 95% Bayesian credible intervals obtained from the Bayesian Hierarchical Spatial model with covariates for each survey year (1993–2014). It is observed in the results for each survey year (1993–2014) that the estimated posterior means for the intercept term *α* differ substantially between the models.

**Table 6:** Summary of posterior means and Bayesian credible intervals of smooth relative risk of neonatal mortality estimated from weighted Bayesian Hierarchical Spatial model using aggregated Ghana demographic and health survey (GDHS) cluster level data.

	Model 1	Model 2	Model 3	Model 4	Model 5
Parameters	Posterior Mean(95% Cr.I)	Posterior Mean(95% Cr.I)	Posterior Mean(95% Cr.I)	Posterior Mean(95% Cr.I)	Posterior Mean(95% Cr.I)
***Fixed Effect***					
*Intercept*	4.64 (-44.67, 53.66)	-2.90 (-4.33,-1.69)	-1.46 (-3.12, -1.42)	-2.73 (-3.95, -1.62)	25.278(-28.39, 78.22)
***individual factors***					
*small body size baby*	0.24(0.05, 0.44)	-0.12(-0.34, 0.08)	0.01(-0.10, 0.12)	-0.02 (-0.20, 0.15)	0.11(0.06, 0.17)
*Median household size*	-0.18(-0.35, -0.02)		-0.10(-0.27, 0.06)	0.04 (-0.13, 0.20)	0.06(-0.17, 0.29)
*median parity*	0.31(0.15, 0.47)	0.25(0.11, 0.38)	0.01(-0.16, 0.18)		0.07(-0.19, 0.31)
*Proportion of poor mothers*	-0.46(-1.20, 0.28)	-0.80(-1.51, -0.11)	-0.99(-1.75, -0.24)		
***Community factors***					
*Number of rural dwellers*	0.10(0.03, 0.16)	0.12(0.07, 0.17)	0.13(0.09, 0.17)	0.15(0.09, 0.20)	0.02(0.001, 0.04)
*Proportion of unimproved sanitation facilities*	0.02(-0.04, 0.09)	-0.03(-0.10, 0.04)	0.001(-0.05, 0.05)	-0.07 (-0.13, -0.01)	0.01(-0.03, 0.04)
*median time to reach water source and return*	-0.004(-0.03, 0.18)	0.01(-0.01, 0.02)	-0.001(-0.02, 0.01)		0.004(-0.02, 0.03)
***Geographical factors***					
*Median Maximum Temperature*	-0.02(-0.19, 0.14)				-0.10(-0.27, 0.08)
*Median Aridity*	0.01(-0.01, 0.04)	0.002(-0.02, 0.03)	-0.009(-0.03, 0.01)	-0.01(-0.04, 0.01)	0.04(0.01, 0.06)
*Median land surface temperature*	-0.01(-0.05, 0.03)	-0.01(-0.04, 0.03)	-0.002(-0.04, 0.04)		
**Random Effect**					
$\tau_{\mu }^{-1}$	-2.25(-4.91, 0.55)	-2.54(-5.05, -2.60)	-2.35 (-3.59,- 1.21)	-0.95 (-2.65, 0.83)	-3.89 (-5.27, -2.49)
$\tau_{v}^{-1}$	2.10(0.38, 4.14)	2.23 (1.08, 3.54)	1.32 (0.21, 2.57)	0.59(-2.00, 3.73)	2.29(1.29, 3.31)
**Model Evaluation Metrics**					
**DIC**	430.94	376.93	483.46	301.93	418.37
**WAIC**	434.00	380.21	494.87	304.31	431.98

Model 1 = Bayesian Hierarchical Spatial model for 1993, Model 2 = Bayesian Hierarchical Spatial model for 1998, Model 3 = Bayesian Hierarchical Spatial model for 2003, Model 4 = Bayesian Hierarchical Spatial model for 2008, Model 5 = Bayesian Hierarchical Spatial model for 2014.

Note: $\tau_{\mu }^{-1}$ and $\tau_{v}^{-1}$ are illustrated on the logarithmic scale.

In 1993, the results of the Bayesian Hierarchical Spatial model in Table 6 showed that a unit increase in the number of small body size babies increased the log relative risk of neonatal deaths [*PM*: 0.24 (95% Crl: 0.05, 0.44)], thus contributed to the observed spatial pattern. When other covariates were controlled in the multivariable model, it is observed that a unit increase in the median household size reduced the risk of neonatal deaths [*PM*: -0.18 (95% Crl: -0.35, -0.02)]. The posterior mean of parity estimated from the model indicated an increase in the risk of neonatal deaths [*PM*: 0.31 (95% Cr.I: 0.15, 0.47)]. On the other hand, the risk of neonatal deaths increased among rural dwellers in the multivariate model [*PM*: 0.10 (95% Cr.I: 0.03, 0.16)]. All the geographical factors used in the model for the 1993 DHS survey year were not significant.

Furthermore, model 2 indicated that a high risk of neonatal deaths was associated with rural dwellers [*PM*: 0.12 (95% Cr.I: 0.07, 0.17)] in 1998. It is also notable in model 2 that an increase in parity was more likely to increase the risk of neonatal deaths [*PM*: 0.25(95% Cr.I: 0.11, 0.38)]. The results in model 3 suggested that a significantly high risk of neonatal deaths was linked to rural dwellers in 2003 GDHS year [*PM*: -0.13 (95% Cr.I: 0.09, 0.17)]. Apparently, babies born to mothers living in rural areas seemed to be more likely to die within 28 days of life [*PM*: 0.15 (95% Cr.I: 0.09, 0.20)] in 2008. It is notable in model 5 that small body size babies increased the risk of neonatal mortality in 2014 [*PM*: 0.11 (95% Cr.I: 0.06, 0.17)]. Additionally, babies born to mothers living in rural areas appeared to have increased risk of neonatal deaths in the 2014 survey year [*PM*:0.02(95%Cr.I: 0.001, 0.04)]. Geographical factor such as aridity contributed significantly to an increased risk of neonatal mortality in 2014 [*Posterior Mean*: 0.04 (95% Cr.I: 0.01, 0.06)].

## Discussion

The current study sought to investigate the spatial and temporal clustering of neonatal mortality across regions and GDHS clusters to inform targeted interventions, especially for the predicted high-risk regions. Our modelling approach is novel, allowing identification of high risk regions for optimal allocation of available limited public health resources where universal intervention is practically impossible. We estimated weighted standardized mortality ratio (SMR) and smooth relative risk (RR) which were mortality risk estimates for each survey year considered in the current study. Results from the exploratory analysis indicated that 1.08, 1.16, 0.95, 0.82, and 1.05 weighted standardized mortality ratios (SMR) and 1.12, 1.02, 0.89, 0.83, and 0.95 smooth weighted relative risks of neonatal mortality were recorded in 1993, 1998, 2003, 2008 and 2014 GDHS years respectively. A glimpse of the weighted SMR and RR revealed a downtrend in the risk of neonatal mortality from 1993 to 2008 but shot up in 2014. Even though SMR provides a vivid and fair idea of the neonatal mortality risk profile of the respective location and year, it was reported in an article [1] that the estimate of SMR could be biased and unreliable if locations with small population sizes are included in the computation. Thus, to address this drawback, Bayesian Hierarchical Spatial and Spatiotemporal models that borrow information from neighboring regions and covariates were considered to smooth out the extreme or rare values associated with a small population size [23].

It is instructive to note that Ashanti and Upper West regions had registered a substantial decline in observed neonatal mortality (weighted) from 1993 to 2014. There was a steady upsurge of neonatal deaths from 1993 to 2014 in the Upper East. Volta and Brong Ahafo regions observed a marginal decline in neonatal deaths from 1998 to 2014. The highest number of neonatal deaths in 2014 was observed in the Upper west region. To observe a meaningful and interpretative trend in neonatal deaths, summary statistics such as weighted neonatal mortality rate was estimated for each survey year. A slight drop in the weighted neonatal mortality rates was observed over time. It is obvious that the policies formulated to reduce child mortality were not very effective to trigger a rapid decline in the neonatal mortality rates. Thus, there is the need to review the policies and focus more attention on the health and well-being of neonates so as to improve their health and in effect reduce neonatal deaths substantially. Many studies using demographic and health survey and other nationally representative datasets arrived at a conclusion on a downward trend in neonatal mortality rates [7,27] contrary to the results from the current study when survey design characteristics were incorporated. However, when we curiously investigated the trend of unweighted observed neonatal deaths, the trend was consistent with findings from previous studies.

Global and local autocorrelation coefficients were computed using Moran’s I statistics for each survey year to examine spatial clustering and spatial heterogeneity using the aggregated GDHS cluster point level data. The global Moran’s I test results rejected the null hypothesis of spatial randomness, thus revealing the existence of spatial clustering of neonatal mortality risk. The existence of spatial autocorrelation suggests that neighboring GDHS clusters have a higher probability to share similar patterns of neonatal deaths than GDHS clusters that are far apart. Even though, the presence of clustering depicts a complete spatial pattern, it does not demonstrate the location of the clusters, Thus, to observe location of clusters, local Moran’s I statistics was computed using local indicator of spatial association (LISA) and the significant results were displayed in a LISA cluster map. On the other hand, the CAR model applied to the regional areal data suggested the existence of spatial clustering.

The Weighted Bayesian Hierarchical Spatiotemporal models with and without covariates were evaluated using model evaluation statistics such as DIC, WAIC, and LPML to select a model that smooth out any inherent noise or extreme values best. The decision on the selection of the best performing model was based on the larger value of LPML which was in favor of the weighted Bayesian Hierarchical Spatiotemporal model. The spatial and temporal random effects components of the best selected model explained the larger amount of variation in the neonatal deaths relative to the other two competing models. Thus, the focus of the geospatial analysis in the current study was centered on the application of weighted Bayesian Hierarchical Spatiotemporal model with covariates which accounts for both spatial and temporal correlation and dependence. The weighted Bayesian Hierarchical Spatiotemporal model with covariates was applied to the regional level aggregated data to estimate smooth relative risk of neonatal mortality and examine the contribution of covariates to spatial clustering and spatiotemporal variations in the risk of neonatal mortality.

The relative risk of neonatal mortality estimated from the weighted Bayesian Hierarchical Spatiotemporal model varied around the overall relative risk of 0.89 with 95% credible interval ranging from 0.11 to 7.61. Additionally, the small body size baby was significant and slightly increased the risk of neonatal mortality holding other covariates constant [Posterior Mean: 0.003 (95% CrI: 0.00,0.01)]. The results from this study is consistent with findings from a previous study [8]. Even though the weighted Bayesian Hierarchical Spatiotemporal model did not explicitly model the causes or factors that influence small body size babies, the literature is replete with information on the possible predictors of small body size babies. Some of the causes of small body size babies included but are not limited to having multiple pregnancies (twins, triplets or more), a problem with the placenta, an infection in the womb, the mother having a chronic health condition, such as high blood pressure or kidney disease, the mother taking certain medicines to treat conditions such as epilepsy and blood clots, a previous pregnancy that resulted in the baby having a low birth weight, smoking, drinking alcohol or using illegal drugs, exposure to air pollution or lead and the baby having a congenital disorder. Thus, health education and promotion would focus activities on controlling and preventing the aforementioned risk factors of small body size babies.

Surprisingly, all the other covariates used in the model were not significant. Apparently, geographical factors such as aridity [Posterior Mean: 0.03 (95% CrI: -0.02, 0.08)] and precipitation [Posterior Mean: 0.01 (95% CrI: -0.003, 0.02)] which were of utmost interest in the current study appeared to increase the risk of neonatal mortality even though they were not significant. A previous study conducted in the United State Of America reported that racial differences in birth weight-specific contributed largely to gaps in neonatal mortality, however, all the covariates considered did not significantly explain variations of the birth weight-specifc mortality to the Black-White neonatal mortality gap [28].

The results of the current study showed that estimation of large values for the posterior means of the precision of spatial random effects were consistent with a statement expressed in a reviewed article that the inclusion of spatial random effect could possibly increase the error variances associated with specific contrast of the data [29,30]. Thus, it is important to indicate that a very large amount of variation in the relative risk of neonatal mortality was attributable to the precision parameters of spatial and temporal random effects. In other words, the precision parameters of both spatial and temporal random effects modeled and controlled the degree of smoothness or extra-Poisson variability in the risk of neonatal mortality. We can draw inference conclusively from the weighted Bayesian Hierarchical Spatiotemporal model results that small body size babies, spatial and temporal random effects contributed to spatial variations and spatial clustering in the risk of neonatal mortality at the regional level.

Even though, a map of observed weighted neonatal mortality rate was produced, it cannot be very accurate in illustrating true spatial variations and spatial clustering because of inherent noise or errors. Nonetheless, it gives fair impression about spatial distribution of neonatal mortality. Generally, the map depicted Spatio-temporal variation and clustering. Thus, we identified a high number of neonatal mortality rate in Western and Northern regions in 1993. The map revealed a low neonatal mortality rate in Upper East region consistently from 1993 to 2014. The Ashanti region recorded a high number of neonatal deaths in 2003, 2008 and 2014. As indicated earlier, we could not rely solely on a map of neonatal mortality rates. To this end, it was imperative to specify an appropriate model that smooths the neonatal data in order to observe true clusters of neonatal deaths and to examine the correlates associated with neonatal mortality clustering. To achieve this, we specified weighted Bayesian Hierarchical Spatiotemporal model which smooths the neonatal data by borrowing information from neighboring regions and accounting for spatial and temporal random effects [21].

Spatial and temporal effects were estimated by weighted Bayesian Hierarchical Spatiotemporal model with covariates. Spatial effects included spatial dependence which was modeled using the CAR model and spatial heterogeneity which was modeled using the *iid* model. On the other hand, temporal effect included structured temporal effect which was modeled using the RW1 model and unstructured temporal effect was modeled using the *iid* model. Furthermore, the inflated posterior mean of the precision of structured spatial and temporal random effect indicated that there was spatial and temporal dependence in the risk of neonatal mortality across the regions overtime. In other words, similar relative risk of neonatal deaths was exhibited by neighboring regions. For instance, spatial clustering or spatial dependence was evident in 1993 where a cluster with nearby regions exhibiting similar risk were identified. A cluster of a high relative risk of neonatal deaths were observed in the Northern, Ashanti, Brong Ahafo, Eastern and Greater Accra regions in 1993. Regions such as Ashanti, Eastern and Northern and Brong Ahafo regions shared similar high risk of neonatal deaths in 1998. Additionally, Northern, Brong Ahafo, Ashanti, Greater Accra, Upper West and Upper East regions which share the same boundary recorded moderately high risk of neonatal deaths in 2003. Geographical areas such as Volta, Eastern and Western regions recorded a low relative risk of neonatal deaths in 2003. Ashanti region is the only geographical area that recorded a relative risk of neonatal dealths above average in 2008. Additionally, Volta and Ashanti regions reported excess risk of neonatal mortality in 2014 and Ashanti region was also identified as a region with elevated risk of neonatal mortality in 1993, 1998, 2008 and 2014. Thus, the Ashanti region should be identified as a priority region of focus as far as neonates’ health care management is concerned. It was indicated in a reviewed article that spatial dependence could be two-dimensional and multidimensional. The same article further explained that an observation of attribute at one location could be correlated with the value of the same attribute at a different location, and the causation pattern could occur in different directions [31].

We also aggregated the data at the GDHS cluster level and applied a weighted Bayesian Hierarchical Spatial model in order to smooth the estimate and identify significant GDHS clusters of the risk of neonatal mortality. Spatial clustering was clearly observed when the relative risk of neonatal mortality obtained from the weighted Bayesian Hierarchical Spatial model was displayed in a LISA cluster map. A LISA cluster map showed that a high-high autocorrelation (hot spot) of the risk of neonatal deaths for DHS clusters were observed in the Northern, Upper West, and some part of Western and Ashanti regions respectively where number of small body size babies, and number of babies born to mothers living in rural areas increased the risk of neonatal mortality in 1993. A high risk of neonatal mortality in one DHS cluster correlated with a high risk of neonatal mortality in neighboring DHS clusters and these were observed in Central, Western, Volta, Brong Ahafo, Eastern and Ashanti regions. Furthermore, a few spatial outliers or overdispersion of the risk of neonatal deaths for DHS clusters were observed in Ashanti, Brong Ahafo and Northern regions where parity and babies born to mothers living in rural areas were significantly associated with a relative risk of neonatal mortality in 1998. It is evident in the LISA cluster map that a significant high–high (hot-spot) risk of neonatal mortality in locations such as DHS clusters was found in Ashanti, Brong Ahafo, and Northern regions where high number of babies born to mothers living in rural areas resulted in a high relative risk of neonatal deaths in 2003. Some DHS clusters were identified as cold-spots in the Upper East and Upper West regions in 2003. Additionally, a GDHS cluster with a high risk of neonatal mortality was surrounded by DHS clusters with a high risk of neonatal mortality in Ashanti, Northern, and the coastal areas while a few GDHS clusters were identified as cold spot in Ashanti and the Northern regions where a high number of babies born to mothers living in rural areas increased the risk of neonatal deaths in 2008. Lastly, it is apparent that a DHS cluster with a significantly high risk of neonatal mortality was surrounded by its neighbors with high risk of neonatal mortality in Ashanti, Eastern and Volta regions in 2014. Conversely, there was a cluster of high risk of neonatal deaths and clusters of low risk of neonatal mortality between DHS clusters in Eastern, Brong Ahafo and Eastern regions in 2014. These findings are very intriguing given that GDHS clusters with elevated risk of neonatal mortality were identified as hot spot areas and should be deemed as priority GDHS clusters. Hence, health resources should be distributed equitably to improve child and maternal health care in order to reduce neonatal deaths to an acceptable level.

It is imperative to note that high-high (hot spot) means that GDHS clusters with above average risk of neonatal mortality share boundaries with neighboring GDHS clusters with above average relative risk of neonatal mortality and also low-low (cold spot) signifies that GDHS clusters with below average relative risk of neonatal mortality share boundaries with neighboring GDHS clutsers with below average relative risk of neonatal mortality. Furthermore, high-low means that GDHS clusters with above average relative risk of neonatal mortality are surrounded by GDHS clusters with low average relative risk of neonatal mortality and finally low-high signifies that GDHS clusters with below average relative risk of neonatal mortality are surrounded by GHDS clusters with above average relative risk of neonatal mortality and low-low (cold spot) autocorrelation give rise to spatial clusters while High-low and low-high give rise to spatial outliers or over dispersion.

We examine correlates associated with the risk of neonatal mortality at the GDHS cluster level for each survey year. The results from the analysis showed that babies born to mothers living in rural areas consistently increased the risk of neonatal mortality in all survey years under consideration. Poverty and rural dwelling were identified by a recent study to have increased the risk of infant mortality which is consistent with the findings from the current study [32]. Thus, rural areas contributed significantly to geographical clustering and an increased relative risk of neonatal mortality. There is the need for government to improve the living conditions of rural dwellers. As indicated in a reviewed literature [33] rural dwellers have less access than urban dwellers to health services, clean water, sanitation and education. Thus, health centers and CHPS compounds should be extended to the rural and hard to reach communities and also health promotion officers should be tasked to sensitize pregnant women and mothers to visit child welfare and Antenatal clinics so that they can benefit substantially from provision of health care services. It is apparent from the results of the current study that a high risk of neonatal mortality was associated with small body size babies in 1993 and 2014.

Furthermore, parity appeared to significantly contribute to a higher relative risk of neonatal mortality in 1993 and 1998. The results from the current study were similar to the results from a previous study [34]. It is important to note that geographical factor such as aridity significantly associated and contributed to spatial variations and spatial clustering of the risk of neonatal mortality in 2014, which is in agreement with a previous study [33].

We wish to state that the main focus of this paper was on spatiotemporal variations in the risk of neonatal mortality. We sought to determine how the distribution of neonatal mortality varies across time and space and this is a classic example of spatial and temporal epidemiology. Improvement of our understanding of spatiotemporal clustering and variation in the risk of neonatal mortality could lead to the development of innovative strategies and targeted interventions for the high risk regions and lessons learnt from the low risk regions which would help in an equitable distribution of scarce health resources such as the provision of health facilities, posting of qualified health professionals to the affected regions, intensifying health education and promotion in the regions with excess risk of neonatal mortality in order to address the menace of neonatal mortality. The application of the hierarchical model identified the regions and DHS clusters with statistically significant high/low neonatal mortality, and this would help in policy formulation to reduce the risk of neonatal mortality.

## Conclusion

The focus of this paper was on quantifying the extent of geospatial clustering of neonatal deaths in Ghana and how they vary over time, and also identifying correlates associated with the observed neonatal mortality clustering. The Moran’s I statistics applied to the aggregated GDHS cluster point level data and CAR model applied to the regional aggregated areal data suggested spatial clustering or spatial dependence in the risk of neonatal mortality. Firstly, we evaluated two models and selected the weighted Bayesian Hierarchical Spatiotemporal model with covariates as the best model to quantify spatial and temporal variations in the risk of neonatal mortality. Thus, we established that the weighted Bayesian Hierarchical Spatiotemporal model with covariates smoothed the data far better than the empty model. Additionally, the map of smooth relative risk of neonatal mortality obtained from the weighted Bayesian Hierarchical Spatiotemporal model illustrated a vivid visualization of spatial clustering and spatial heterogeneity. Furthermore, the Northern, Greater Accra, and Ashanti regions were identified as the most consistent hot-spot regions for the entire period under consideration. Most importantly, the LISA cluster map identified DHS clusters with elevated risk (hot-spots) of neonatal mortality.

The key model findings that could be acted upon included the identification of hot spot regions in Ghana such as the Northern, Greater Accra, and Ashanti regions. This means that more of the scarce resources should be allocated to these hotspot regions. Thus, Greater Accra, Northern and Ashanti regions should be priority regions of focus as far as neonates’ health care management is concerned. And also for all the covariates included in the model at the regional level, only small body size babies significantly contributed to the spatiotemporal clustering and variation in the risk of neonatal mortality. There is a need to focus attention on controlling and preventing possible risk factors of small body size babies. On the other hand, some GDHS clusters were identified as hot spot and cold spot areas. Thus, the hot spot areas will be the GDHS clusters of focus or priority GDHS clusters. The study also found that babies born to mothers living in rural areas, small body size babies, parity and aridity significantly associated and contributed to spatial variations and spatial clustering of the risk of neonatal mortality.

In summary, the results of the current study shed more light on or expand our understanding of spatial clustering and spatiotemoporal variations in the risk of neonatal mortality in Ghana. The findings are also very crucial in identifying the regions and GDHS clusters within the regions with an elevated risk of neonatal mortality and possibly provide health managers with insightful information to distribute the scarce health resources and interventions equitably in order to tackle the menace of neonatal mortality and by extension improve child health.

## Supporting information

S1 Appendix Letter from DHS authorizing the use of GPS data (Request for Geographic Data - wistaal@gmail.com - Gmail.pdf). (PDF)

S2 Appendix Letter from DHS authorizing the use DHS datasets (DHS Download Account Application - wistaal@gmail.com - Gmail.pdf). (PDF)
