# Supplementary material for: Spatio-temporal variations in neonatal mortality rates in Ghana: An application of hierarchical Bayesian methods
Source: PLOS Glob Public Health. 2022 Sep 8;2(9):e0000649. doi: 10.1371/journal.pgph.0000649 (PMC10021147; doi:10.1371/journal.pgph.0000649)
Supplement: S1 Appendix — (PDF) [file pgph.0000649.s001.pdf]

Request for Geographic Data

Inbox

gpsrequests@dhsprogram.com via amazonses.com

Tue, 8 Feb, 08:44

to me

Your application for access to the GPS data has been approved. Please note that the user agreement states that you will not share the data with others, unless their name, email address and institutional affiliation are included in your request.

To ensure respondent confidentiality, we randomly displace the GPS latitude/longitude positions for all DHS, MIS, and AIS surveys. The GPS data for SPA surveys is not displaced.
The displacement for all DHS, MIS, and AIS surveys is randomly carried out so that:
-Urban clusters are displaced up to 2 kilometers.
-Rural clusters are displaced up to 5 kilometers, with 1% of the rural clusters displaced up to 10 kilometers.

The displacement is restricted so that the points stay within the country and within the DHS survey region. In surveys released since 2009 the displacement is restricted to the country's second administrative level wherever possible. Each of the datasets for surveys released since 2012 comes with a "GPS_Displacement_README" document which states the level at which the displacement was restricted for that particular survey.

The displacement introduces random error, which can substantively affect the results of analyses that look at small geographic areas. Specifically, measuring direct distance from a GPS location to some other site (facility, school, etc.) is NOT appropriate.

For more information about how to use the GPS datasets, you may wish to consult the recent publications in our Spatial Analysis Reports series:

- Geographic Displacement Procedure and Georeferenced Data Release Policy for the Demographic and Health Surveys (English) http://dhsprogram.com/publications/publication-SAR7-Spatial-Analysis-Reports.cfm

- Guidelines on the Use of DHS GPS Data (English) http://dhsprogram.com/publications/publication-SAR8-Spatial-Analysis-Reports.cfm

- Spatial Interpolation with Demographic and Health Survey Data: Key Considerations (English) http://dhsprogram.com/publications/publication-SAR9-Spatial-Analysis-Reports.cfm

- Linking Geospatial Data from Facility and Household Surveys To Inform Health Program Decision-Making (English) http://dhsprogram.com/publications/publication-SAR10-Spatial-Analysis-Reports.cfm

Refer to the "DHS_README" file in each data package for explanations of variables in the dataset, the DHS GPS data format can also be found at: http://www.dhsprogram.com/What-We-Do/upload/MEASURE-DHS-GPS-Data-Format.pdf

You may view the documentation for GPS data at http://www.dhsprogram.com/What-We-Do/GIS.cfm and http://www.dhsprogram.com/faq.cfm. The DHS user forum (http://userforum.dhsprogram.com/) also contains information about DHS geographic data.

If you are a first time user of DHS GPS data, please view The DHS Program Spatial Data and Resources video at http://youtu.be/VJlsuccJGYE. The Spatial Data Repository
(http://spatialdata.dhsprogram.com/resources/) also contains video tutorials about the website, map gallery, DHS survey region boundaries, and downloading and opening geographically linked national or sub-national DHS indicator data.

If you intend to link GPS data to external covariate datasets for further analysis, consider using the geospatial covariate data files prepared by The DHS Program: http://spatialdata.dhsprogram.com/covariates/

Login at https://dhsprogram.com/data/dataset_admin/login_main.cfm to download the geographic data.

If you have further questions please visit The DHS Program User forum (http://userforum.dhsprogram.com/) or contact us at gpsrequests@dhsprogram.com.

Thank you.

LOGIN INFORMATION:
Login Email: wistaal@gmail.com
Password: (use password selected when you registered)
